# MOVE MORE, GAIN LESS: EFFECT OF A RECREATIONAL TRAIL SYSTEM ON CHILDHOOD BMI

**DOI:** 10.1111/coep.12448

**Published:** 2019-10-31

**Authors:** Bongkyun Kim, Michael R. Thomsen, Rodolfo M. Nayga, Di Fang, Anthony Goudie

**Affiliations:** ^1^ Korea Research Institute for Local Administration Seoul the Republic of Korea; ^2^ Agricultural Economics and Agribusiness University of Arkansas Fayetteville AR 72701; ^3^ Arkansas Center for Health Little Rock AR 72201

## Abstract

We investigate the effect of residential proximity to recreational trails on childhood body mass index (BMI). We find that children living within 0.5 miles of recreational trails have BMI *z*‐scores that are 0.0412 to 0.0507 standard deviations lower than those who do not live within 0.5 miles of trails. We also find that living nearby trails reduces the probability of becoming obese by 1.6 percentage points. The impact for BMI *z*‐score is larger for children qualifying for free or reduced‐price meals and for Hispanic children. These findings suggest that improving neighborhood amenities conducive to physical activity may help reduce disparities in childhood obesity. (*JEL* I10, R10)

**ABBREVIATIONS**
ACHI: Arkansas Center for Health Improvement
BMI: Body Mass Index
CDC: Centers for Disease Control and Prevention
NAICS: North American Industry Classification System
NHTS: National Household Travel Survey
PE: Physical Education
SD: Standard Deviations

## I. INTRODUCTION

Childhood obesity is an important public health issue in the United States that impacts long‐term health and economic outcomes. For example, there is growing evidence that obesity at a young age may lead to lower educational attainment (Black, Johnston, and Peeters 2015; Ding et al. 2009; Sabia 2007), lower earnings (Cawley 2004), and fewer employment opportunities (Lindeboom, Lundborg, and van der Klaauw 2010; Morris 2007; Rooth 2009). Obese children are more likely to be obese adolescents and adults (Cunningham, Kramer, and Narayan 2014; Serdula et al. 1993). A variety of policies have been implemented to reduce the childhood obesity rate, primarily through school‐based interventions, but additional efforts at the community level are also needed.

Relative to other interventions, improvements in the built environment have small negative spillovers and high political feasibility (Sandy et al. 2013). For instance, school‐level interventions often involve mandatory rules that could restrict choices of healthy‐weight children without significant benefit. On the other hand, improvements in the built environment could benefit both overweight and normal weight children without restricting choice. Because improvements to the built environment are available to both the affluent and less affluent (at least at the point of consumption), investments in the built environment may be a less controversial way to reduce health inequalities.

In this paper, we investigate the effect of residential access to recreational trails, a specific component of the built environment, on childhood body mass index (BMI) and obesity rates. Proximity to recreational trails is measured by the distance from the centroid of the census block of the child's residence to the nearest trail. The study makes use of a unique dataset that links measured BMI of children to geocoded trails. Findings indicate that children who live within 0.5 miles of recreational trails have BMI *z*‐scores that are 0.0412 standard deviations (SD) lower than those who live beyond 0.5 miles of the trails. When children living within 0.5 miles of the trails are compared to children living beyond a mile of the trails, the beneficial effect increases to the 0.0507 standard deviations. We also find that living within a half mile of a trail reduced the probability of being obese by 1.6 percentage points and the probability of being overweight or obese by 2.0 percentage points. These estimates are similar across subsamples of majority and minority children and across subsamples comprised of lower‐ and higher‐income children.

In comparison to the overweight or obese classifications, BMI *z*‐score is a more granular measure of the child's weight status. We find a larger beneficial impact of trail proximity on the BMI *z*‐scores of Hispanic children (−0.0585 SD) relative to non‐Hispanic, white children (−0.0327 SD). Hispanics constitute the predominant minority group in the study region. Disproportionate impacts on BMI *z*‐score were also found for lower‐income children (−0.0622 SD) as indicated by their eligibility for free or reduced‐price school meals relative to higher‐income children (−0.0228 SD). These findings imply that improving neighborhood amenities through recreational trails could play a role in preventing excess weight gain among at‐risk groups of children and reduce disparities in childhood BMI. For context, these impacts on BMI *z*‐score are roughly equivalent to a 1‐pound decrease in the weight of a 10‐year‐old boy of average height. While this may seem small, the effect is within the range of effects estimated from other school‐based interventions (Cawley, Frisvold, and Meyerhoefer 2013; Cawley, Meyerhoefer, and Newhouse 2007; Millimet, Tchernis, and Husain 2010).

The potential endogeneity of trail locations is the main challenge for identification. There are two possible sources of endogeneity. First, the location of recreational trails might be affected by political influence so that routes could favor socioeconomically advantaged groups. Second, awareness of the benefits of trails may influence the choice of residential location. To the extent that unobserved characteristics of these households, such as preferences for health or investment in children's well‐being, impact children's weight in the same direction as the proximity to trails, results would overestimate the true benefits of trail proximity.

We address these issues in several ways. In terms of recreational trail locations, we obtained background information indicating that trails expanded along small creeks or streams during the study period. These stream paths presented a low‐cost and efficient way of implementing the trail system, which reduces concerns of political influence in trail locations. To address endogenous sorting by households, we analyze samples of nonmoving and moving households separately. We find some evidence of positive selection on observables among children whose households moved to residences nearby trails. Because the sample covers the period during which the trails were being built, trail proximity is more likely to be exogenous for those who do not change residential location during the sample period. Moreover, the estimated effects for children from moving households are smaller and less significant than those for children from non‐moving households. Children from moving households who live nearby the trails are less likely to be eligible for free or reduced‐price school meals. This implies that our results are not driven by households from advantaged backgrounds. In addition, we conduct a number of sensitivity tests. The results are robust to changes in cutoff points used to define the proximity to a trail and to changes in other model specifications. A falsification test using planned recreational trails allows us to rule out the possibility that findings are being driven by endogenous sorting of nonmoving households before the trails were built. Finally, a permutation test supports the idea that the estimated effects are from living nearby actual trail locations. In absence of a randomized field experiment or a valid instrumental variable for trail proximity, these results contribute to existing evidence that access to recreational trails can help address the childhood obesity problem and contribute more broadly to a growing literature on the effect of the built environment on body weight among children and adults.

A growing number of studies examine the effect of the built environment in economics, but a majority of these have focused on the effect of the commercial food environment (Alviola et al. 2014; Anderson and Matsa 2011; Chen, Florax, and Snyder 2013; Courtemanche and Carden 2011; Currie et al. 2010; Davis and Carpenter 2009; Dunn, Sharkey, and Horel 2012; Powell 2009; Thomsen et al. 2016). Among the few studies looking at the effect of neighborhood amenities are Fan and Jin (2014) and Sandy et al. (2013). Fan and Jin (2014) show that children who live in neighborhoods with parks or playgrounds have a lower BMI and lower risk of being overweight or obese compared to children in neighborhoods without parks or playgrounds. Using data for children in Indianapolis, Sandy et al. (2013) find that the presence of recreational trails near the home significantly reduces children's weight and that the effect is mainly driven by children living in safe neighborhoods. Our study builds on Sandy et al. (2013), but differs in the following respects. First, Sandy et al. (2013) analyze children in Indianapolis, a major U.S. city. Our data differ in that they represent a less urbanized population covering several communities in Northwest Arkansas.^1^ Second, Sandy et al. (2013) use BMI data from clinical visits. The data used in this study are drawn from a statewide BMI screening program covering all public schoolchildren in Arkansas. To the extent that children who visited clinics have different characteristics from the population of children at large, our data provide a valuable addition to Sandy et al. (2013)'s findings because they represent the entire population.^2^ Third, we use a variety of measures for trail proximity. Although our results are robust to the proximity measures used in Sandy et al. (2013), other measures may be more appropriate for Northwest Arkansas, which is less densely populated than Indianapolis. Lastly, and as noted above, we assess the sensitivity of our findings through several additional analyses.

The remainder of the paper proceeds as follows. In Section II, we provide detailed information on the data used in this paper. The empirical strategy is laid out in Section III. The results are presented in Section IV. Section V explains and reports robustness checks of our main results. Sections VI and VII contain a discussion of key findings and conclusions, respectively.

## II. DATA

Three datasets are used in this study. The first contains longitudinal information from Arkansas' statewide BMI screening program of public schoolchildren and is housed at the Arkansas Center for Health Improvement (ACHI). The second consists of trail records from the Northwest Arkansas Planning Commission. The third contains business listings from ReferenceUSA®. Using these data, it is possible to compile a detailed picture of children's BMI with respect to trail locations, other features of the built environment, and the commercial food environment. Each dataset is described in turn.

### A. *Arkansas Childhood BMI Data*

The Arkansas General Assembly passed Act 1220 of 2003. With this act, Arkansas became the first in the United States to mandate measurement of weight and height of public schoolchildren.^3^ The BMI measurements started in the 2003/2004 school year. Since that time, public schoolchildren in kindergarten through 10th grade have been measured biennially.^4^ School participation rates in the BMI assessment program are high, 94–99% (Justus et al. 2007). Raw BMI measures were converted to age and sex specific *z*‐scores and percentiles using the Centers for Disease Control and Prevention (CDC) reference growth charts (CDC 2015).

Research staff at ACHI geocoded children's addresses and assigned them to the 2010 U.S. Census block. We were unable to use exact residential locations given confidentiality concerns. This may be one source of potential bias, and the choice of census blocks as the geographic unit was made to minimize this bias. The census block is the smallest geographic unit maintained by the U.S. Census Bureau. The centroid of the census block is used as an approximation of the child's residential location. There are over 186 thousand census blocks in Arkansas, 5,457 of which fall within the communities that comprise our study region. The physical size of these census blocks is small. Each block contains only a few children in our sample in any given year.^5^ Regardless of whether block centroids or rooftop locations are used in measuring distances to trails, there is some degree of ambiguity as to the route taken to a trail because foot or bike traffic may not necessarily follow the network of roadways surrounding the home. As described in the next section, we explore distances from the block centroid to the trail using both radial and networked distances.

Along with the BMI and household location, the data set contains demographic information about the children and indicates whether a child qualifies for free or reduced‐price school meals based on household income. We use longitudinal BMI data including the 2003/2004 through the 2014/2015 school years for our primary analysis. One of the advantages of the Arkansas BMI dataset is that BMI is objectively measured by trained personnel using standardized equipment and measurement protocols. This provides BMI measures that are free of the common biases that occur when self‐reported BMI is used.

### B. *Trail Data in Northwest Arkansas*

**Figure 1:**
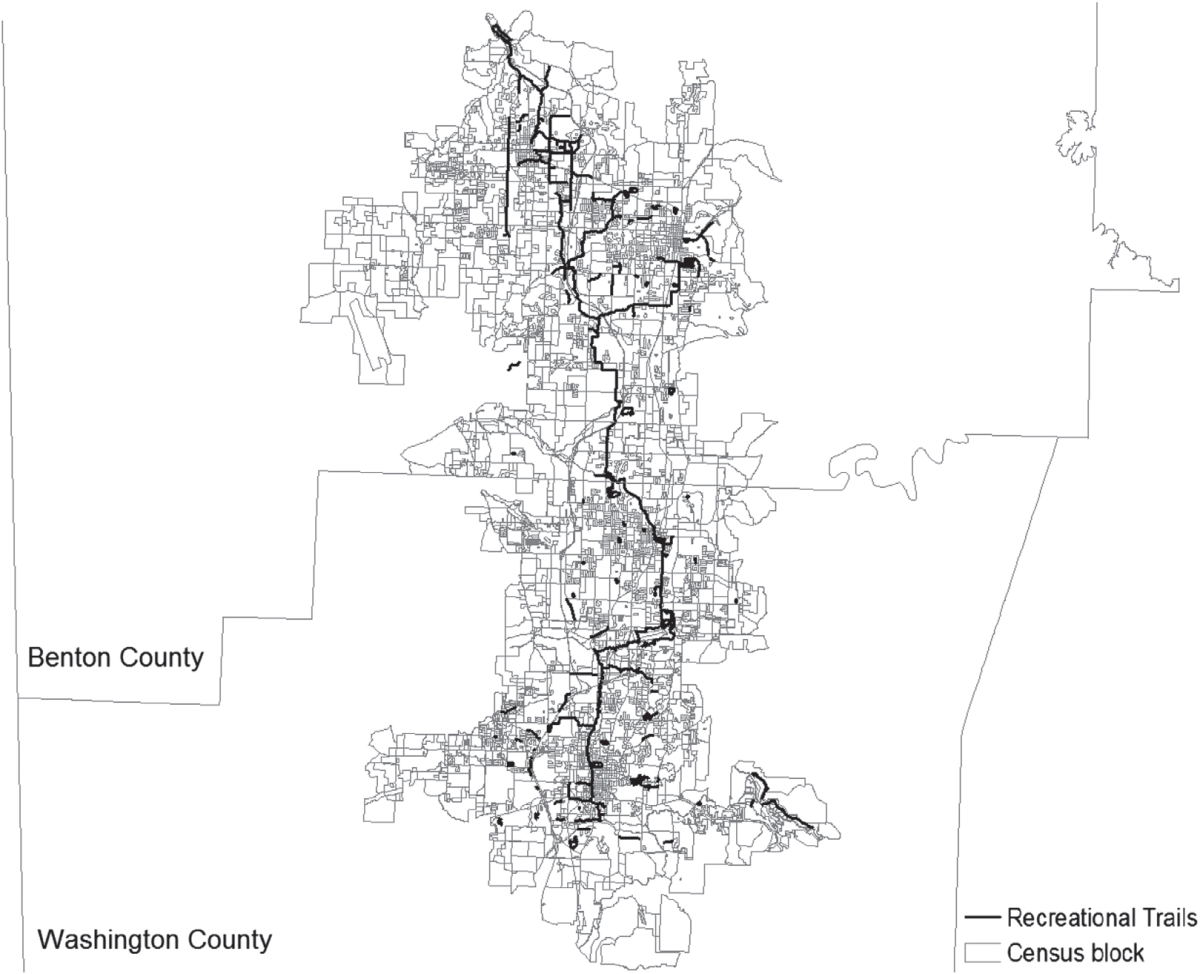
The Recreational Trail System in Northwest Arkansas

As of 2016, approximately 234 km of recreational trails extended across Benton and Washington counties in Arkansas (i.e., Northwest Arkansas). Figure 1 shows the location of these trails. In the initial stages of construction, the project plan required much of the trail system to follow roadways. However, the focus changed to an off‐road trail system after 2009.^6^ The route of the off‐road system was discussed through a series of community workshops, but a key consideration in deciding the route was proximity to a local creek or stream. Streams provided an advantage as trails could make use of existing bridge systems with some box culverts being converted for use as trailways beneath roadways. The fact that trails were mainly built along streams considerably mitigates the concern about endogenous trail locations (Sandy et al. 2013). Most of creeks or streams that the trails follow are first or second order streams based on National Hydrography Dataset classification. First‐order streams are the smallest permanent streams and second‐order streams result when two first‐order streams combine (Benke and Cushing 2005). In our study region, these streams do not afford meaningful recreational opportunities aside from their convenience as trail routes.

**Figure 2:**
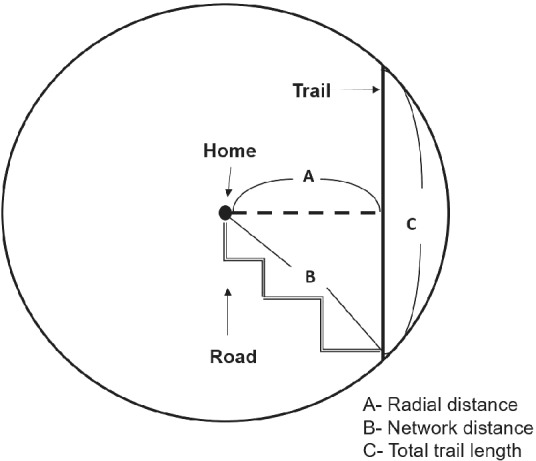
Description of Distance Measurements

Several local governments in Northwest Arkansas provided data sources for measuring trail locations. Three different binary measures are used to measure trail access. The first indicates presence of a trail within a radial distance of 0.5 miles (about 800 m) from the centroid of the census block of the child's residence. The second indicates the presence of a tail within a networked distance of 0.5 miles. This reflects the distance between the centroid of the census block and a trail access point along the network of roadways. Finally, following Sandy et al. (2013), a third measure is based on the total length of trails within a circle with 0.5 mile radius and centered on the census‐block centroid. For this third measure, the indicator takes a value of 1 if the total length of trails within the circle is greater than the average length (about 380 m). Figure 2 provides a visual representation of these three different measures. The 0.5‐mile radius chosen for this study differs from the 0.25‐mile radius used by Sandy et al. (2013). We use a larger radius because our analysis focuses on a less urbanized area. In addition, recent studies have shown that acceptable walking distance is greater than 0.25 miles. Based on the 2009 National Household Travel Survey (NHTS), Yang and Diez‐Roux (2012) showed that mean walking distance for recreational purposes is about 0.5 miles. Kim (2015) found that pedestrians in the San Francisco Bay Area walk about 0.35 miles on average. In our primary analysis, we focus on major trails, those that form or connect to the backbone of the trail system. We do, however, assess sensitivity of findings to the inclusion/exclusion of minor trails.

### C. *Data on Establishment Locations*

Verified locations of businesses and establishments were obtained from the ReferenceUSA® database for each year of the study. Establishments were classified using the North American Industry Classification System (NAICS) code along with business name and other descriptors available to identify characteristics of the establishment.

Establishments were classified into the following: chain fast‐food restaurants, liquor stores, pawnshops, bars, child and youth services centers, and recreational sport centers. Fast‐food restaurants have been recognized as a factor affecting childhood obesity rates in previous studies (Alviola et al. 2014; Anderson and Matsa 2011; Chen, Florax, and Snyder 2013; Currie et al. 2010; Davis and Carpenter 2009; Dunn, Sharkey, and Horel 2012; Powell 2009). According to previous studies (Borrell, Graham, and Joseph 2016; Lovasi et al. 2013; Lumeng et al. 2006; Sandy et al. 2013), neighborhood safety is one of the factors affecting physical activity. Following the criminology literature (Groff and Lockwood 2014; McCord et al. 2007), liquor stores, pawnshops, and bars are used as indicators of higher‐crime areas. Child and youth services centers are defined as establishments providing nonresidential social assistance services for children and youth. We control for these centers because they may be a source of information or programming to promote healthy behaviors. Lastly, playgrounds, parks, and recreation facilities are cited as the main neighborhood amenities for children's physical activities aside from recreational trails (Corder et al. 2011; Grow et al. 2008). Thus, together with the nearest distance to park or playground, the estimations include controls for recreational sport centers. The number of these centers is calculated within 0.5 miles of the centroid of the child's census block of residence.

Table 1 presents descriptive statistics for the sample. In this table, summary statistics are presented for all children as well as for children in moving and nonmoving households. The average BMI *z*‐score is 0.7034 for children in moving households, whereas it is 0.5934 for children in nonmoving households. The proportion of children who are classified as overweight or obese, and obese is also slightly larger among movers in comparison to nonmovers. While the likelihood of living nearby a trail is similar for both types of children, movers are more likely to be from lower‐income or minority households. For example, there are significantly more non‐White children (12.6 percentage points) and children qualifying for free or reduced‐price meals (22.2 percentage points) among the sample of moving households. Finally, the neighborhoods where movers live have more chain fast‐food restaurants and more of the aforementioned establishments associated with higher‐crime areas.

**Table 1:** Summary Statistics

	(1)	(2)	(3)
	Movers	Nonmovers	Full Sample
BMI *z*‐score	0.7034	0.5934	0.6391
	(1.0649)	(1.0651)	(1.0664)
Indicator for overweight or obese	0.3926	0.3484	0.3668
	(0.4883)	(0.4765)	(0.4819)
Indicator for obese	0.2122	0.1783	0.1924
	(0.4089)	(0.3827)	(0.3942)
Indicator for a trail within 1/2 mile of home using radial distance	0.2822	0.2912	0.2875
	(0.4501)	(0.4543)	(0.4526)
Indicator for a trail within 1/2 mile of home using network distance	0.1482	0.1526	0.1508
	(0.3553)	(0.3596)	(0.3578)
Indicator for greater than avg. total length of trails within 1/2 mile of home	0.2165	0.2261	0.2221
	(0.4119)	(0.4183)	(0.4157)
Indicator for male	0.5153	0.5140	0.5145
	(0.4998)	(0.4998)	(0.4998)
Indicator for female	0.4847	0.4860	0.4855
	(0.4998)	(0.4998)	(0.4998)
Indicator for Asian	0.0796	0.0566	0.0661
	(0.2707)	(0.2310)	(0.2485)
Indicator for white	0.4823	0.6087	0.5562
	(0.4997)	(0.4881)	(0.4968)
Indicator for African‐American	0.0529	0.0286	0.0387
	(0.2239)	(0.1667)	(0.1929)
Indicator for Hispanic	0.3738	0.2925	0.3263
	(0.4838)	(0.4549)	(0.4688)
Indicator for other races	0.0114	0.0137	0.0127
	(0.1063)	(0.1161)	(0.1122)
Age (years)	9.9892	9.7959	9.8762
	(3.2003)	(3.5314)	(3.3979)
Indicator for free or reduced‐price meals	0.6414	0.4212	0.5127
	(0.4796)	(0.4938)	(0.4998)
Indicator for full price meal	0.3586	0.5788	0.4873
	(0.4796)	(0.4938)	(0.4998)
Number of chain fast‐foods within 1/2 mile from home	0.5697	0.4421	0.4951
	(1.4354)	(1.2446)	(1.3286)
Number of liquor stores within 1/2 mile from home	0.1169	0.0934	0.1032
	(0.3540)	(0.3331)	(0.3421)
Number of pawnshops within 1/2 mile from home	0.1759	0.1295	0.1488
	(0.5267)	(0.4541)	(0.4861)
Number of bars within 1/2 mile from home	0.1273	0.0968	0.1094
	(0.5950)	(0.5767)	(0.5845)
Number of child and youth services centers within 1/2 mile from home	0.2538	0.2218	0.2351
	(0.7862)	(0.7403)	(0.7599)
Number of recreational sport centers within 1/2 mile from home	0.1091	0.0844	0.0946
	(0.3677)	(0.3185)	(0.3400)
Distance to the nearest park or playground (km)	1.2646	1.3704	1.3264
	(0.9565)	(1.1423)	(1.0703)
*N*	76,198	107,302	183,500

*Note*: Mean values are represented. Standard deviations are in parentheses.

## III. EMPIRICAL STRATEGY

In order to examine how residential proximity to recreational trails affects childhood BMI, two different specifications are estimated. The first is specified in Equation (1):

(1) $$\begin{array}{cc} {\text{Outcome}}_{\mathrm{ibt}} & =\alpha_{0}+\alpha_{1}I{\left(\text{Dist}1/2\right)}_{\mathrm{ibt}}\\ & \;+\alpha_{2}X_{\mathrm{ibt}}+\delta_{b}+\gamma_{t}+\epsilon_{\mathrm{ibt}} \end{array}$$

where *i* indicates the child, *b* indicates the residential census block group, and *t* is the year of BMI measurement. Three outcome variables are considered: the BMI *z*‐score, an indicator for whether the child's *z*‐score fell above the 85th percentile on the age‐sex specific CDC growth charts (overweight or obese), and an indicator for whether the child's *z*‐score was above the 95th percentile on these reference growth charts (obese). As already noted, *I(Dist1/2)* is an indicator for whether the radial distance from the residential census block centroid to a major trail is less than 0.5 miles. *X* is a vector of controls for individual and neighborhood characteristics including sex, race, age, age squared, an indicator of free or reduced‐price meal status, number of years living within 0.5 miles of a trail, (number of) chain fast‐food restaurants, youth centers, recreational sport centers, liquor stores, pawnshops, bars, and distance to the nearest park or playground.^7^ *X* also includes an indicator for free or reduced‐price meal status. This reflects the child's household income level. Children from households with incomes at or below 130% of the federal poverty level qualify for free school meals. Children from families with income between 130% and 185% of the federal poverty level are eligible for reduced‐price meals. The distance to the nearest park or playground is the radial distance from the residential block centroid to the nearest park or playground. Finally, *X* includes the total number years that the child lived within a half mile of a trail. Block‐group and year fixed effects are represented by *δ* and *γ*, respectively. Robust standard errors are clustered by census block.^8^ For our analysis, we only include observations with a BMI *z*‐score between ±4 as this excludes biologically unlikely values. The coefficient of interest in Equation (1) is *α*_1_, which shows the effect of living within a half‐mile of the trail system compared to living beyond a half‐mile of the trail system.

In our primary specification, we use the radial distance as our measure of trail proximity. This is because 559 out of 5,457 census block centroids in our sample are not on the road network. Thus, there would be a significant number of observations with missing values if we used a network distance measure. Moreover, because our study region is less urbanized, using the total length of trails within a circle of the centroid of the census block as used in Sandy et al. (2013) may not properly represent the proximity to the trails.^9^ As noted above, we also increase the distance threshold to 0.5 miles as opposed to 0.25 miles as used in Sandy et al. (2013). Block group fixed effects are used because inclusion of individual fixed effect results in the loss of a large number of observations due to the fact that there is little variation in the trail proximity indicator, *I(Dist1/2)*, within individuals over the sample period.^10^ Approximately 72% of the individuals did not experience a change in trail proximity status. Because block groups encompass several census blocks, the inclusion of block‐group fixed effects permits us to control for time‐invariant differences in neighborhood characteristics while still preserving within‐neighborhood variation in the trail proximity measure.

In addition to Equation (1), we estimate a specification similar to that used by Currie et al. (2010) in their study on the effect of proximity to fast‐food restaurants on childhood obesity. The second model we estimate is:

(2) $$\begin{array}{cc} {\text{Outcome}}_{\mathrm{ibt}} & =\beta_{0}+\beta_{1}I{\left(\text{Dist}1/2\right)}_{\mathrm{ibt}}\\ & \;+\beta_{2}I{\left(\text{Dist}2/3\right)}_{\mathrm{ibt}}+\beta_{3}I{\left(\text{Dist}1\right)}_{\mathrm{ibt}}\\ & \;+\beta_{4}X_{\mathrm{ibt}}+\delta_{b}+\gamma_{t}+\epsilon_{\mathrm{ibt}} \end{array}$$

where *I(Dist2/3)*, and *I(Dist1)* are indicators for whether the radial distance from the centroid of the census block of residence to a trail is less than 2/3 miles (about 1,072 m), and is less than 1 mile (about 1,600 m), respectively. The definitions of other variables are as in Equation (1). The coefficient on the 0.5 miles indicator (the 2/3 miles indicator) represents the difference between the effect of living within 0.5 miles (within 2/3 miles) of recreational trails and the effect of living within 2/3 miles (within 1 mile) of the trails. Of primary interest in Equation (2) is the sum of the three coefficients on the proximity indicators (*β*_1_ + *β*_2_ + *β*_3_), which represents the effect of living within a half mile of a trail compared to living with no trails within a mile radius of the residence. In this specification, the identification assumption is that in absence of recreational trails, children living within 0.5 miles from the trails and children living within 2/3 miles from the trails are assumed to have a similar change in BMI. This estimation diminished the impact of unobservable differences between the groups by comparing children who are at a slightly different distance to a trail (Currie et al. 2010). Note that while the coefficient of interest in Equation (1) shows the benefit of living 0.5 miles from the trails relative to living beyond 0.5 mile, the coefficient of interest in Equation (2) represents the benefit of living 0.5 miles from the trails relative to living beyond a mile from the trails. Although they do not reflect the exact same effects, they are comparable.

## IV. RESULTS

### A. *Primary Results*

Table 2 presents the effect of trails on childhood BMI *z*‐score and weight status. Estimates from the specification in Equation (1) with the 0.5 mile indicator of trail proximity are in panel A. Panel B presents estimates from the specification with multiple proximity indicators (Equation (2)). Columns (1)–(3) present estimates with BMI *z*‐score as the outcome variable. Columns (4)–(6) contain estimates for probability of being overweight or obese. Finally, estimates for the probability of being obese are presented in columns (7)–(9). For each of the three outcomes, estimates are presented from the full sample and for samples containing only nonmovers and only movers.

**Table 2:** The Effect of Trail Proximity on Childhood BMI and Weight Status

	BMI *z*‐score			Overweight or obese			Obese		
	(1)	(2)	(3)	(4)	(5)	(6)	(7)	(8)	(9)
	Full Sample	Nonmovers	Movers	Full Sample	Nonmovers	Movers	Full Samples	Nonmovers	Movers
Panel A Model with single proximity indicator									
Living within	−0.0412***	−0.0545***	−0.0268	−0.0200***	−0.0246***	−0.0170**	−0.0161***	−0.0203***	−0.0132**
1/2 miles of a trail	(0.0126)	(0.0169)	(0.0176)	(0.0052)	(0.0071)	(0.0079)	(0.0043)	(0.0057)	(0.0063)
*N*	183,500	107,302	76,198	183,500	107,302	76,198	183,500	107,302	76,198
*R* ^2^	0.054	0.059	0.047	0.046	0.052	0.038	0.045	0.052	0.039
Panel B Model with multiple proximity indicators									
Living within	−0.0113	−0.0400*	0.0162	−0.0003	−0.0115	0.0100	−0.0063	−0.0126	−0.0007
1/2 miles of a trail	(0.0165)	(0.0220)	(0.0234)	(0.0076)	(0.0099)	(0.0107)	(0.0056)	(0.0079)	(0.0083)
Living within	−0.0183	0.0007	−0.0405	−0.0190**	−0.0098	−0.0319***	−0.0052	−0.0016	−0.0105
2/3 miles of a trail	(0.0167)	(0.0215)	(0.0248)	(0.0081)	(0.0104)	(0.0111)	(0.0059)	(0.0077)	(0.0087)
Living within	−0.0211	−0.0188	−0.0241	−0.0069	−0.0056	−0.0086	−0.0094**	−0.0086	−0.0108
1 mile of a trail	(0.0135)	(0.0177)	(0.0171)	(0.0057)	(0.0072)	(0.0077)	(0.0047)	(0.0058)	(0.0067)
Sum of coefficient	−0.0507***	−0.4181***	−0.0484**	−0.0262***	−0.0269***	−0.0305***	−0.0209***	−0.0228***	−0.0220***
(*p*‐value)	(.0021)	(.0065)	(.0293)	(.0001)	(.0024)	(.0025)	(.0002)	(.0013)	(.0080)
*N*	183,500	107,302	76,198	183,500	107,302	76,198	183,500	107,302	76,198
*R* ^2^	0.054	0.059	0.048	0.046	0.053	0.038	0.046	0.052	0.039

*Notes*: Robust standard errors, clustered by census block, are in parentheses. Models include block‐group fixed effects.

* *p* < .1,

** *p* < .05,

*** *p* < .01.

Estimates presented in column (1) of panel A indicate that children living within 0.5 miles of a trail have *z*‐scores that are 0.0412 SD lower than children who live beyond 0.5 miles of a trail. The sum of the three coefficients in Equation (2) in column (1) of panel B indicates that children living within 0.5 miles of a trail have a *z*‐score that is 0.0507 SD lower than children who live beyond a mile from a trail. A comparison of this sum with the estimate in panel A (beyond one mile versus beyond 0.5 miles) shows that the two findings are similar. Finally, as shown in the first row of panel B, there is no significant effect of living within 0.5 miles of a trail beyond living within 2/3 miles of a trail.

The results in column (4) and column (7) of panel A show that living within 0.5 miles of a trail reduces the probability of becoming overweight or obese, and of becoming obese by 2.0 percentage points and 1.6 percentage points, respectively. Much like the results when BMI *z*‐score is the outcome, the sum of the three coefficients in panel B is statistically significant. The magnitude of this sum is comparable to the estimate shown in panel A.

### B. *Movers vs. Nonmovers*

One potential factor threatening the validity of these findings is endogenous sorting (e.g., if households with advantaged backgrounds choose residential locations near trails). To explore this issue, we first examine whether there is evidence of selection on observables between moving households and nonmoving households with respect to trail proximity. This is done by regressing race and school meal status on the trail proximity variable in Equation (1).

As shown in Table A1 of Appendix A, there is no evidence of selection on observables among nonmovers. However, among movers, there is a significantly higher number of white children and a significantly lower number of non‐White children and children qualifying for free and reduced‐price meals who live within 0.5 miles of the trails. Findings are suspect if socioeconomically advantaged households are better able to invest in children's well‐being. To the extent that unobserved factors also reinforce a healthy weight status, our findings shown in column (1) of Table 2 might overestimate the true effects if children from moving households primarily drive the estimated beneficial trail effect.

Column (2) and (3), of Table 2 alleviate this concern to some degree. The trail effect is larger in magnitude for the nonmovers. In panel A, the coefficient for nonmovers is −0.0545 and is significant at the 1% level, while the coefficient for movers is −0.0268 and is insignificant. Similarly, the bottom of column (2) and column (3) in panel B show larger effects of trail proximity for nonmovers relative to movers. This same dominance in the effect among nonmovers is also observed in columns (5)–(6) and (8)–(9). These results provide evidence that estimates from the combined sample are not being influenced disproportionately by endogenous sorting of families from more advantaged socioeconomic backgrounds. One possible explanation for smaller effects among movers compared with nonmovers is that children from advantaged backgrounds may have less time to use recreational trails and may engage in physical activities through paid facilities and privately organized physical activities (e.g., participation in youth sports leagues or dance classes). Alternatively, if families who move into a new area are not familiar with the local environment, parents may be reluctant to allow their children to use the trails due to safety concerns. In sum, the results in Table 2 show that living nearby recreational trails has a significant effect on reducing children's weight, and these effects are not mainly caused by unobservable characteristics of socioeconomically advantaged household.^11^ Because results from both Equations (1) and (2) are similar, but the interpretation of Equation (1) is more intuitive, the remainder of the paper will report trail effects estimated from Equation (1).

### C. *Heterogeneous Effects*

To check if there is heterogeneity in trail effects across subpopulations, we consider subsamples by gender, grade, race, school meal status, and length of time living nearby the trails. Dividing the sample by length of exposure can capture cumulative effects. Specifically, we consider children living within 0.5 miles of a trail for 5 years or more separately from children living within 0.5 miles of a trail for 4 years or less.

As shown in Table 3, the trail effect is significant and negative for both boys and girls. While the magnitude of the effect on BMI *z*‐score in panel A is slightly larger for boys, the magnitude of the effect on the probability of being overweight or obese, and for being obese in panel B and panel C is similar across genders. The results in columns (3)–(4) show that the trail effect is more important among younger children and suggests that younger children may be behind the overall effect presented in Table 2. This finding is consistent with evidence from a report on regional trail usage showing that a majority of bicycle users in our study area are adults with young children (The Walton Family Foundation 2018).

**Table 3:** The Effect of Trail Proximity on Childhood BMI and Weight Status: Sub‐Sample Results

		(1)	(2)	(3)	(4)	(5)	(6)
		Male	Female	Kinder., 2nd, 4th grades	6th, 8th, 10th grades	Free/reduced‐price meals	Full‐price meals
Panel A							
BMI *z*‐score	Living within	−0.0455***	−0.0384**	−0.0567***	−0.0103	−0.0622***	−0.0228
	1/2 miles of a trail	(0.0171)	(0.0158)	(0.0153)	(0.0180)	(0.0173)	(0.0172)
*N*		94,416	89,084	102,804	80,696	94,076	89,424
*R* ^2^		0.058	0.049	0.057	0.055	0.031	0.035
Panel B							
Overweight or obese	Living within	−0.0203***	−0.0198***	−0.0293***	−0.0030	−0.0177**	−0.0231***
	1/2 miles of a trail	(0.0072)	(0.0067)	(0.0063)	(0.0082)	(0.0073)	(0.0074)
*N*		94,416	89,084	102,804	80,696	94,076	89,424
*R* ^2^		0.052	0.041	0.047	0.047	0.028	0.031
Panel C							
Obese	Living within	−0.0160***	−0.0165***	−0.0230***	−0.0046	−0.0174***	−0.0166***
	1/2 miles of a trail	(0.0059)	(0.0054)	(0.0050)	(0.0063)	(0.0065)	(0.0050)
*N*		94,416	89,084	102,804	80,696	94,076	89,424
*R* ^2^		0.052	0.037	0.049	0.045	0.027	0.033

		(7)	(8)	(9)	(10)	(11)
		White	Hispanic	Other races	5 years or more	4 years or less
Panel A						
BMI *z*‐score	Living within	−0.0327**	−0.0585**	−0.0320	−0.0711**	−0.0325**
	1/2 miles of a trail	(0.0164)	(0.0228)	(0.0371)	(0.0299)	(0.0135)
*N*		102,060	59,869	21,571	29,202	154,298
*R* ^2^		0.026	0.020	0.045	0.062	0.055
Panel B						
Overweight or obese	Living within	−0.0187***	−0.0200**	−0.0278*	−0.0336***	−0.0163***
	1/2 miles of a trail	(0.00678)	(0.0100)	(0.0155)	(0.0118)	(0.0058)
*N*		102,060	59,869	21,571	29,202	154,298
*R* ^2^		0.024	0.020	0.032	0.058	0.046
Panel C						
Obese	Living within	−0.0113**	−0.0196**	−0.0318***	−0.0195*	−0.0137***
	1/2 miles of a trail	(0.0051)	(0.0088)	(0.0118)	(0.0102)	(0.0047)
*N*		102,060	59,869	21,571	29,202	154,298
*R* ^2^		0.027	0.021	0.030	0.060	0.045

*Note*: The subsamples in columns (10) and (11) contain children living within 0.5 miles of trails for 5 years or more or 4 years or less respectively. Robust standard errors, clustered by census block, are in parentheses.

* *p* < .1,

** *p* < .05,

*** *p* < .01.

More importantly, in terms of the BMI *z*‐score in panel A, results in columns (5)–(9) show that the effect of trails for lower‐income children who qualify for free or reduced‐price meals and Hispanic children are significantly larger in magnitude than the effects for white children and more affluent children who do not qualify for school meal discounts, respectively. However, as shown in panel B and panel C, we do not find particularly larger effects for probability of being overweight or obese, and for being obese across ethnicity or school meal status. The BMI *z*‐score indicates the child's BMI relative to the reference population whereas the overweight or obese and obese indicators show whether the child's BMI is in an unhealthy range. Thus, these findings suggest that while the beneficial effects of trails on the likelihood of an unhealthy weight is similar across socioeconomically advantaged and disadvantaged groups, there may be stronger benefits among socioeconomically disadvantaged groups in preventing excess weight gain among children in the normal weight range. In this respect, the larger beneficial effects on BMI *z*‐score among lower‐income children and Hispanic children provides some evidence that public investment in the built environment can reduce health inequalities. This finding is consistent with ancillary evidence that trail usage is, in fact, higher in low‐income areas of Northwest Arkansas (The Walton Family Foundation 2015). The low monetary cost of enjoying recreational trails once they have been established might explain the differential usage of the trails among higher and lower‐income households. Lastly, as shown in columns (10)–(11), the effects of the trails are larger for children who have lived nearby trails for a longer period of time, suggesting that cumulative effects exist.

We also examine if there is heterogeneity in the effects by presence of establishments correlated with neighborhood safety. Since we do not have geographically detailed crime data in Northwest Arkansas during the sample period, the numbers of crime‐associated establishments, as described in Section II, within 0.5 miles from the centroid of the census block of the child's residence are used as a proxy for neighborhood safety. These establishment counts are interacted with the trail proximity indicator in Equation (1). As shown in Table 4, the only significant estimate is the effect of pawnshops on the probability of being overweight or obese in panel B. The coefficient on this interaction term is positive, which indicates the trail effect is smaller in areas with more pawnshops. However, in most of cases, we do not find significant interaction effects with these establishments.

**Table 4:** The Effect of Trail Proximity on Childhood BMI and Weight Status: Heterogeneous Effects by Presence of Crime‐Associated Businesses

**Panel A BMI *z*‐score**	
Living within 1/2 miles of a trail	−0.0400***
	(0.0134)
Living within 1/2 miles of a trail × Liquor stores	−0.0187
	(0.0243)
Living within 1/2 miles of a trail × Pawnshops	0.0181
	(0.0163)
Living within 1/2 miles of a trail × Bars	−0.0176
	(0.0143)
*N*	183,500
*R* ^2^	0.054
**Panel B Overweight or obese**	
Living within 1/2 miles of a trail	−0.0201***
	(0.00554)
Living within 1/2 miles of a trail × Liquor stores	−0.0106
	(0.0103)
Living within 1/2 miles of a trail × Pawnshops	0.0122*
	(0.0074)
Living within 1/2 miles of a trail × Bars	−0.0066
	(0.0057)
*N*	183,500
*R* ^2^	0.046
**Panel C obese**	
Living within 1/2 miles of a trail	−0.0167***
	(0.0045)
Living within 1/2 miles of a trail × Liquor stores	−0.0058
	(0.0084)
Living within 1/2 miles of a trail × Pawnshops	0.0076
	(0.0057)
Living within 1/2 miles of a trail × Bars	−0.0005
	(0.0050)
*N*	183,500
*R* ^2^	0.045

*Note*: Robust standard errors, clustered by census block, are in parentheses.

* *p* < .1,

^**^ *p* < .05,

*** *p* < .01.

## V. ROBUSTNESS CHECKS

### A. *Changes in Specification*

We have shown a significant trail effect on BMI *z*‐score in our primary analysis. As a next step, we test whether these results are sensitive to specification changes. First, given that the proximity indicator used to obtain the results reported above is from the major trails which form or connect to the backbone of the two‐county trail system, we re‐estimate the model using all trails including other multi‐purpose trails. These other trails are short and sometimes isolated. Next, we assess whether results are robust to different cutoff points for the trail proximity indicator. Finally, we include a county specific time trend in the model to control for unobserved factors in the error term that might have the same trend as children's BMI status.

Results of these robustness checks are shown in Table 5. When proximity is measured relative to all trails in column (1), the effects are smaller but are still significant at the 1% level. This finding suggests that most physical activities occur along the major trails. Results in columns (2) and (3) represent different cutoff points for determining trail access. Although the coefficients from a decreased cutoff point to 1/3 miles are insignificant for BMI *z*‐score and probability of obesity, and smaller in magnitude than our baseline results, they continue to be negative. The trail‐exposed sample is much smaller at this cutoff. Only 17% of the children in our sample are within 1/3 miles of trails. In contrast, the coefficients from an increased cutoff point to 2/3 miles continues to be significant at the 1% level, but shows a slightly smaller magnitude of effects for BMI *z*‐score and probability of obesity relative to results using the baseline cutoff of 1/2 mile. Lastly, as shown in column (4), the inclusion of a county specific time trend has a minimal impact on the main results. Overall, results in Table 5 show that the primary results in Table 2 are robust to changes in model specification.

**Table 5:** The Effect of Trail Proximity on Childhood BMI and Weight Status: Robustness and Specification Tests

		(1)	(2)	(3)	(4)
		All Trails	1/3 mile Cutoff	2/3 mile Cutoff	County Specific Time Trend
Panel A					
BMI *z*‐score	Living within	−0.0319***	−0.0195	−0.0332***	−0.0413***
	1/2 miles of a trail	(0.0116)	(0.0135)	(0.0124)	(0.0126)
*N*		183,500	183,500	183,500	183,500
*R* ^2^		0.054	0.054	0.054	0.054
Panel B					
Overweight or obese	Living within	−0.0157***	−0.0101*	−0.0210***	−0.0200***
	1/2 miles of a trail	(0.0049)	(0.0054)	(0.0054)	(0.0052)
*N*		183,500	183,500	183,500	183,500
*R* ^2^		0.046	0.046	0.046	0.046
Panel C					
Obese	Living within	−0.0108***	−0.0072	−0.0123***	−0.0162***
	1/2 miles of a trail	(0.0040)	(0.0046)	(0.0043)	(0.0043)
*N*		183,500	183,500	183,500	183,500
*R* ^2^		0.045	0.045	0.045	0.045

*Note*: Robust standard errors, clustered by census block, are in parentheses.

* *p* < .1,

^**^ *p* < .05,

*** *p* < .01.

### B. *Falsification Tests*

**Figure 3:**
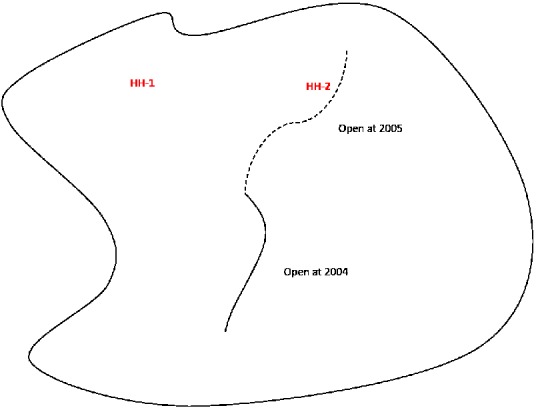
Example of Falsification Test Using Planned Trails

We conduct two simple falsification tests. As a first test, following Courtemanche and Carden (2011), we use children's height as a dependent variable to check whether the effect is actually from a decrease in weight. Although we showed that the primary results are not driven by moving children, it is still possible that some nonmoving households obtained information on the trail routes and relocated before the children from these households were observed in the study. In order to check this possibility, as a second falsification test, we exploit the fact that segments of the trail system were built at different time periods. A specific example is shown in Figure 3. In this figure, the solid line indicates a trail segment that opened in 2004 while the dashed line indicates a trail segment that opened in 2005. A child in household 1 (HH‐1) of the figure lives far from the trail in both 2004 and 2005. A child in household 2 (HH‐2) lives far from the trail in 2004, but lives close to the trail in 2005. If proximity to trails drives our findings, we should not find a significant effect between the 2004 BMI *z*‐score of the child living in HH‐2 and the 2005 trail proximity indicator. Operationalizing this example more generally, we restrict samples to children: (1) who did not live within 0.5 miles of the trails at time *t*‐1, but lived within 0.5 miles of the trails at time *t*, and (2) who never lived within 0.5 miles of the trails both at time *t*‐1 and time *t*. We then regress the average of BMI *z*‐scores at *t*‐1 on the indicator for living nearby trails at time *t*. If we find significant effects from the test, it could be evidence that households who have stronger preference for children's health, but did not change their residence during our study periods, sorted to be nearby the trails even before the trails were built.

The results are shown in Table 6. In column (1) where children's height is used as a dependent variable, we do not find significant results. In column (2) where we exploit planned trails, the effects of the proximity indicator before trails are built are insignificant for BMI *z*‐score and for probability of overweight or obesity in panel A and panel B. However, in panel C, the coefficient for probability of obesity is significant at the 10% level with a negative sign. This result suggests the possibility of residential sorting among nonmoving households who have extreme weight status before we observed the data for our study. While the coefficient is marginally significant, and the results for BMI *z*‐score and for overweight or obesity are insignificant, we acknowledge that results should be interpreted with this potential bias in mind.

**Table 6:** The Effect of Trail Proximity on Childhood BMI and Weight Status: Falsification Tests

		(1)	(2)
		Height	Planned Trails
Panel A			
BMI *z*‐score	Living within	−0.0326	−0.0194
	1/2 miles of a trail	(0.0346)	(0.0333)
*N*		183,500	46,566
*R* ^2^		0.867	0.080
Panel B			
Overweight or obese	Living within	–	−0.0141
	1/2 miles of a trail		(0.0144)
*N*			46,566
*R* ^2^			0.071
Panel C			
Obese	Living within	–	−0.0179*
	1/2 miles of a trail		(0.0107)
*N*			46,566
*R* ^2^			0.071

*Note*: Robust standard errors, clustered by census block, are in parentheses.

* *p* < .1,

^**^ *p* < .05,

^***^ *p* < .01.

### C. *Placebo Tests*

So far, we have assumed that the mechanism driving our main results is that living nearby recreational trails allows children to be more engaged in outdoor activities. Although our findings are robust to changes in model specification and falsification tests, it is still possible that the estimated effects are merely from sampling variation. In other words, if we can find significantly larger effects (in absolute value terms) from several hypothetical trail locations, it could cast doubt on the proximity to trails as a primary factor in decreasing childhood BMI. In order to check this possibility, we conduct a placebo test by arbitrarily moving the trail locations but not the children's locations and re‐estimating the model. The idea of this test is to compare the estimated effect from the true trail location to the distribution of estimates obtained using false trail locations and then check where the true estimated effect falls within the empirical distribution of the placebo effects. For a credible result, the estimated effect from actual trail locations should be larger than most of the effects that are estimated using the hypothetical trail locations. For this placebo test, we move the trail locations by 0.1 miles in easterly and westerly directions from the actual trails up to 5 miles. In each case, the primary model is estimated as if the hypothetical trail locations were the true trail locations. We limit the changes up to 5 miles in both directions from the actual trails because a majority of people in Northwest Arkansas live within this boundary.

**Figure 4:**
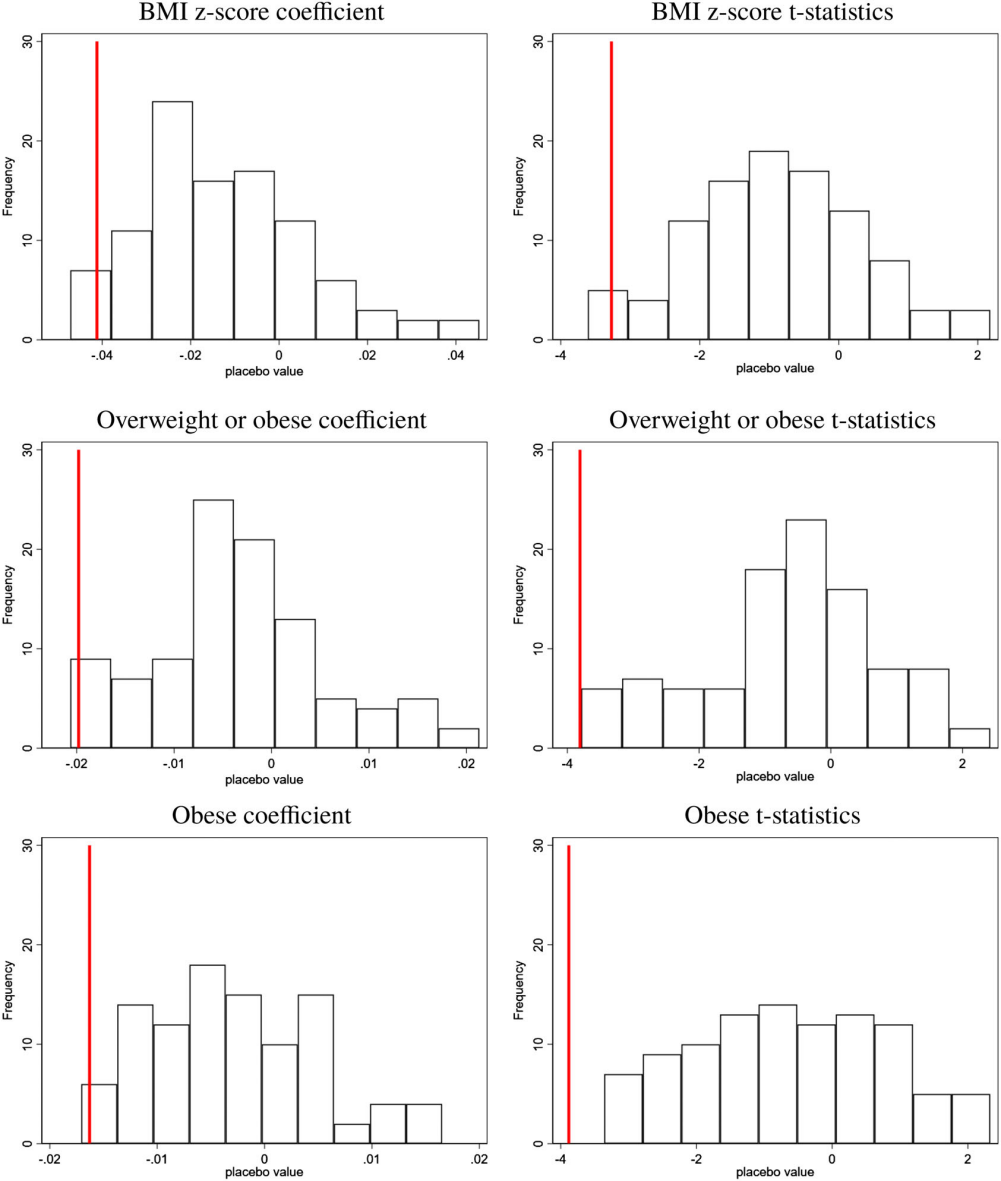
Placebo Tests: Moving Location of Trails

Figure 4 presents the results. In both panels, the vertical line indicates the value estimated using the actual trail location. Of the hundred estimated results, the estimate from actual trail location is larger than all but five estimated effects from the hypothetical locations.^12^ The t‐statistic from the actual trail location is also much larger than that obtained from the placebo locations. In short, the results of the placebo test support the notion that increases in physical activity from living close to the trails is the main mechanism that explains the observed decreases in childhood BMI.^13^

## VI. DISCUSSION

The key finding is that proximity to recreational trails is associated with lower BMI *z*‐scores. One remaining question is whether the effect is economically significant. To address this question, consider what this estimated effect means in terms of a decrease in pounds (kilograms). Our sample mean BMI *z*‐score for boys is 0.6830 which corresponds to the 75th BMI percentile. The estimated effect from our model in column (1) of Table 3 is −0.0455, which suggests that access to trails could reduce this *z*‐score to 0.6375 (=0.6830–0.0455). This decrease would move the average boy in our sample from the 75th to the 74th BMI percentile. According to CDC growth charts, a 10‐year‐old boy at the 75th percentile with average height (4.54 ft [138.5 cm]) would weigh 77.16 pounds (35.0 kg). With the same height, the weight at the 74th percentile is 76.72 pounds (34.8 kg) to 76.94 pounds (34.9 kg). Thus, the estimated effect of our finding is identical to a 0.22 pounds (0.1 kg) to 0.44 pounds (0.2 kg) decrease in weight. The weight decrease based on the estimate from the subsample of Hispanic children, and of children qualifying for free or reduced‐price meals is equivalent to 0.22 pounds (0.1 kg) and 1.10 pounds (0.5 kg), respectively.

Although it is impossible to directly compare the magnitude of effects across studies due to differences in empirical strategy, sample, and study periods, these magnitudes are not negligible compared to the effects estimated from other studies that focused on environmental factors thought to impact childhood obesity. For example, Thomsen et al. (2016) find that children living in food desert neighborhood have about 0.5 pounds higher weights on average compared to children staying in nonfood desert neighborhood. School district policy pertaining to placing Arkansas preteens in middle or elementary schools amounted to about a 1 pound decrease in the weight (Zeng et al. 2016).^14^ When compared with Cawley, Frisvold, and Meyerhoefer (2013)'s study, which estimates a 9.6% decrease in BMI *z*‐score of 5th grade children from 60 min/week of physical education (PE) classes in the United States,^15^ our results are equivalent to the effect of approximately 30 min/week of additional PE class time. Lastly, when taking into account the evidence of limited effect from school‐based interventions, such as the National School Lunch Program, or reducing soda access in school, (Millimet, Tchernis, and Husain 2010; Taber et al. 2014), we argue that the findings from our study on the impact of trails is of practical significance.

## VII. CONCLUSION

In this paper, we investigated the relationship between living nearby a recreational trail and children's BMI status. We used a unique panel dataset that contains children's BMI information from 2004/2005 through the 2014/2015 school year in Arkansas. This was merged with information about trails, the food environment, and the built environment during the same time period. Findings indicate that living nearby recreational trails is associated with lower childhood BMI and obesity rates. Our estimation shows that children living within 0.5 miles of the trails have BMI *z*‐scores that are about 0.0412 standard deviations lower than those who do not live within 0.5 miles of the trails. The estimated beneficial effect increases to the 0.057 standard deviations when we compare children living within 0.5 miles of a trail with children living beyond a mile of the trail. In particular, we found the effect of trails to be the strongest among lower income children, those whose families qualify for free or reduced‐price meals and Hispanic children at −0.0622 and −0.0585, respectively. These effects are equivalent to approximately 0.22 to 1.10 pounds difference in weight. We also found that the probability of being overweight or obese, and of being obese is lower by 2.0 percentage points, and 1.6 percentage points, respectively, on average. In spite of the modest magnitude, the effect of this study is not negligible compared with results from other studies examining environmental impacts on childhood body weight. Since childhood obesity is particularly problematic in certain racial or ethnic groups,^16^ and minority groups comprise some of the fastest growing population segments, our findings provide evidence that improving neighborhood amenities could be an effective strategy to reduce disparities in childhood obesity rates.

There are several limitations of this study. First, the dataset only contains information on public schoolchildren and findings may not generalize to children in private schools or children who are home‐schooled. Second, while our main findings are driven by children from nonmoving households whose families are less likely to have endogenously chosen to live near recreational trails, we acknowledge that potential confounders cannot be completely ruled out. For example, there might still exist differences in unobservable characteristics between households living within 0.5 miles from the trails and households living beyond 0.5 miles from the trails among nonmoving households or that unobservable characteristics might change over time. The marginally significant coefficient for the probability of being obese in the falsification tests might reflect this possibility. Therefore, without a valid quasi‐experiment, our results may not represent a direct causal link between living nearby trails and children's BMI. Third, even though we found that the effects are significant for some subgroups and the placebo test suggests that increased physical activity using the trails is a possible reason for the main findings of the study, we are unable to more deeply examine the direct mechanisms behind these findings with our data. Further formal investigation will be required. Finally, while the sample in this study is comprised of less urbanized population in Northwest Arkansas compared to sample from Indianapolis in Sandy et al. (2013), our study area was becoming more urbanized during the period of study and most of the census blocks in our study area were classified as urban blocks. Urbanization is receiving more attention as a factor in childhood obesity (Ogden et al. 2018). Examining heterogeneous effects by rural and urban areas, however, should be investigated when relevant data are available. Lastly, given increasing interest in the long‐term consequences of childhood obesity and improvement in the built environment, future research looking beyond children's weight status to other outcomes, such as academic achievement, would be valuable.

## Footnotes

1 The four largest communities in our study region are Fayetteville, Springdale, Rogers, and Bentonville, Arkansas with populations (2010 Census) of 73,580, 69,797, 55,994, and 35,301, respectively. In contrast, the population of Indianapolis was 820,445. Population densities (2010 Census) ranged from 1,128 persons per square mile in Bentonville to 1,670 persons per square mile in Springdale. The population density in Indianapolis was 2,264 persons per square mile.

2 In fact, Sandy et al. (2013) found that the data they used included a higher number of low‐income and minority households compared to the demographics of their study area.

3 As of the 2018–2019 school year, approximately 85% of schools in Arkansas were public schools.

4 During the early years of the program, all children were assessed each year. Beginning in the 2006/2007 academic year, only children in even‐numbered grades were assessed. For consistency across time, we include only children in kindergarten and even‐numbered grades through 10th grade.

5 The average size of the census blocks in our study area is approximately 340 m × 340 m. On average there are about two children per block, per year during the study period.

6 About 74% of the trail length was built after 2009.

7 As shown in Table 1, since whites and Hispanics are the major racial and ethnic groups in Northwest Arkansas, an “Other races” category is used to indicate children not in one of these two groups.

8 Results are robust to clustering of standard errors at the school level.

9 We show the results using total length of trails and network distance in table A2 of the appendix.

10 The relatively short number of panel years for many of the children is the main factor that limits within‐individual variation. Approximately 71% of individuals are in the panel three or fewer times.

11 We further classify moving households in terms of those moving nearby trails and those moving far from trails to investigate heterogeneous effects by direction of the move. However, we do not find any significant differences among them.

12 Hypothetical locations with larger coefficient than the actual trail location are for those eastbound by 0.4–0.8 mile. The trail system itself extends north to south as shown in Figure 1. Thus easterly and westerly permutations should best reflect whether the trails themselves matter or whether the findings presented above reflect sampling variation. However, we found similar results when we alter the placebo test by moving trail locations southerly and northerly.

13 Unfortunately, we do not have data on the number of trail users. However, as of 2015, the number of cyclist and pedestrians per 1,000 population on the trails in our study is 4.45 and 4.40, respectively. These usage rates are similar to that of San Francisco (for cyclists) and San Diego County (for pedestrians), which are considered as some of the most walkable cities in the United States (The Walton Family Foundation 2015). Past studies have also found that proximity to parks or recreational facilities is associated with increase in physical activities (Kaczynski and Henderson 2007).

14 This study reports the results only in *z*‐scores. We calculated weight decrease in pounds from their estimates using the CDC growth charts.

15 Note that while results in Cawley, Frisvold, and Meyerhoefer (2013) represent treatment on the treated effects, our results show intent to treat because we do not know which children used the trails. Typically, intent to treat studies finds smaller effect sizes than treatment on the treated effects.

16 For example, 22.8% of Hispanics and 24.4% of non‐Hispanic black females aged between 12 and 19 years old were obese in 2011–2014 (Ogden et al. 2016).

## APPENDIX A 1

**Table A1:** Regression of Observable Characteristics on Trail Proximity

	(1)	(2)	(3)	(4)
	White	Hispanic	Other Races	Free/Reduced‐Price Meal
**Panel A: Nonmovers**				
Living nearby trails	−0.0368	−0.0350	−0.0019	−0.0067
	(0.0227)	(0.0236)	(0.0069)	(0.0244)
*N*	107,302	107,302	107,302	107,302
*R* ^2^	0.013	0.006	0.007	0.012
**Panel B: Movers**				
Living nearby trails	0.1160***	−0.0853***	−0.0304***	−0.0512**
	(0.0216)	(0.0219)	(0.0010)	(0.0212)
*N*	76,198	76,198	76,198	76,198
*R* ^2^	0.016	0.007	0.006	0.007

*Note*: Robust standard errors, clustered by census block, are in parentheses.

^*^ *p* < .1,

** *p* < .05,

*** *p* < .01.

**Table A2:** The Effect of Proximity to the Trails on Children's Weight Status Using Alternative Distance Measurements

		BMI *z*‐score			Overweight or obese			Obese		
		(1)	(2)	(3)	(4)	(5)	(6)	(7)	(8)	(9)
		Whole Sample	Nonmovers	Movers	Whole Sample	Nonmovers	Movers	Whole Sample	Nonmovers	Movers
Panel A										
Single distance indicator model using total length of trails	Living within	−0.0257*	−0.0552***	0.0057	−0.0131**	−0.0236***	−0.0037	−0.0103**	−0.0155***	−0.0080
	1/2 miles of a trail	(0.0135)	(0.0179)	(0.0192)	(0.0055)	(0.0073)	(0.0085)	(0.0045)	(0.0060)	(0.0070)
*N*		183,500	107,302	76,198	183,500	107,302	76,198	183,500	107,302	76,198
*R* ^2^		0.054	0.059	0.047	0.046	0.052	0.038	0.045	0.052	0.039
Panel B										
Single distance indicator model using network distance	Living within	−0.0541***	−0.0736***	−0.0418**	−0.0219***	−0.0268***	−0.0214**	−0.0144***	−0.0157**	−0.0181**
	1/2 miles of a trail	(0.0145)	(0.0200)	(0.0213)	(0.0058)	(0.0083)	(0.0091)	(0.0050)	(0.0064)	(0.0080)
*N*		174,257	101,681	72,576	174,257	101,681	72,576	174,257	101,681	72,576
*R* ^2^		0.054	0.059	0.047	0.046	0.052	0.038	0.046	0.052	0.039
Panel C										
Multiple distance indicator model using network distance	Living within	−0.0355	−0.0575**	−0.0290	−0.0110	−0.0153	−0.0133	−0.0045	−0.0035	−0.0107
	1/2 miles of a trail	(0.0220)	(0.0290)	(0.0297)	(0.0091)	(0.0123)	(0.0131)	(0.0078)	(0.0096)	(0.0107)
	Living within	0.0099	0.0147	0.0103	0.0072	0.0045	0.0111	−0.0022	−0.0018	−0.0029
	2/3 miles of a trail	(0.0226)	(0.0289)	(0.0302)	(0.0094)	(0.0121)	(0.0133)	(0.0081)	(0.0102)	(0.0107)
	Living within	−0.0245*	−0.0220	−0.0292	−0.0208***	−0.0162*	−0.0269***	−0.0045	−0.0075	−0.0017
	1 mile of a trail	(0.0147)	(0.0196)	(0.0202)	(0.0062)	(0.0085)	(0.0091)	(0.0054)	(0.0070)	(0.0077)
	Sum of coefficient	−0.0501***	−0.0648***	−0.0479**	−0.0246***	−0.0270***	−0.0291***	−0.0112**	−0.0128*	−0.0153*
	(*p*‐value)	(.0029)	(.0037)	(.0450)	(.0002)	(.0032)	(.0050)	(.0493)	(.0756)	(.0819)
*N*		174,257	101,681	72,576	174,257	101,681	72,576	174,257	101,681	72,576
*R* ^2^		0.054	0.059	0.048	0.046	0.052	0.039	0.046	0.052	0.039

*Note*: Total length of trails in panel A is indicator variable for 1 if the total distance is greater than average total length. Robust standard errors, clustered by census block, are in parentheses.

* *p* < .1,

** *p* < .05,

*** *p* < .01.
